# Evaluation in Cameroon of a Novel, Simplified Methodology to Assist Molecular Microbiological Analysis of *V*. *cholerae* in Resource-Limited Settings

**DOI:** 10.1371/journal.pntd.0004307

**Published:** 2016-01-06

**Authors:** Amanda K. Debes, Jerome Ateudjieu, Etiene Guenou, Anna Lena Lopez, Mark Philip Bugayong, Pearl Joy Retiban, Marcelino Garrine, Inacio Mandomando, Shan Li, O. Colin Stine, David A. Sack

**Affiliations:** 1 Johns Hopkins University Bloomberg School of Public Health, Baltimore, Maryland, United States of America; 2 Department of Biomedical Sciences, University of Dschang, Dschang, Cameroon; 3 M.A. SANTE (Meilleur Accès aux Soins de Santé), Yaoundé, Cameroon; 4 Division of Health Operations Research, Ministry of Public Health, Yaoundé, Cameroon; 5 University of the Philippines Manila–National Institutes of Health, Manila, Philippines; 6 Research Institute for Tropical Medicine, Muntinlupa, Philippines; 7 Centro de Investigação em Saúde de Manhiça (CISM), Maputo, Mozambique; 8 Instituto Nacional de Saúde (INS), Ministério da Saúde, Maputo, Mozambique; 9 University of Maryland School of Medicine, Baltimore, Maryland, United States of America; Massachusetts General Hospital, UNITED STATES

## Abstract

**Background:**

*Vibrio cholerae* is endemic in South Asia and Africa where outbreaks of cholera occur widely and are particularly associated with poverty and poor sanitation. Knowledge of the genetic diversity of toxigenic *V*. *cholerae* isolates, particularly in Africa, remains scarce. The constraints in improving this understanding is not only the lack of regular cholera disease surveillance, but also the lack of laboratory capabilities in endemic countries to preserve, store and ship isolates in a timely manner. We evaluated the use of simplified sample preservation methods for molecular characterization using multi-locus variable-number tandem-repeat analysis (MLVA) for differentiation of V*ibrio cholerae* genotypes.

**Methods and Findings:**

Forty-seven *V*. *cholerae* isolates and 18 enriched clinical specimens (e.g. stool specimens after enrichment in broth) from cholera outbreaks in Cameroon were preserved on Whatman filter paper for DNA extraction. The samples were collected from two geographically distinct outbreaks in the Far North of Cameroon (FNC) in June 2014 and October 2014. In addition, a convenience sample of 14 isolates from the Philippines and 8 from Mozambique were analyzed. All 87 DNAs were successfully analyzed including 16 paired samples, one a cultured isolate and the other the enriched specimen from which the isolate was collected. Genotypic results were identical between 15 enriched specimens and their culture isolates and the other pair differed at single locus. Two closely related, but distinct clonal complexes were identified among the Cameroonian specimens from 2014.

**Conclusions:**

Collecting *V*. *cholerae* using simplified laboratory methods in remote and low-resource settings allows for subsequent advanced molecular characterization of *V*. *cholerae* O1. These simplified DNA preservation methods identify *V*. *cholerae* and make possible timely information regarding the genetic diversity of *V*. *cholerae*; our results set the stage for continued molecular epidemiological research to better understand the transmission and dissemination of *V*. *cholerae* in Africa and elsewhere worldwide.

## Author Summary

Cholera, caused by the bacterium *Vibrio cholerae*, causes an estimated 3–5 million cases every year and more than 100,000 deaths. The highest disease burden is reported from Africa, however, the epidemic potential and transmission patterns among circulating strains is scarcely understood. The challenges of disease surveillance are constrained by the costs associated with laboratory confirmation and sample preservation. To improve the ability to identify the cholera disease burden, to subsequently understand the molecular epidemiology of circulating cholera strains, and to elucidate transmission patterns, we applied simplified collection methodologies to facilitate timely molecular characterization of *V*. *cholerae* isolates from Cameroon, Mozambique and the Philippines. Enriched specimens as well as cultured isolates were examined, demonstrating that enriched specimens provide sufficient material for genetic analysis. The results of the genetic analyses did not suggest significant genetic diversity within two distinct outbreaks in Cameroon. The study detected a possible relationship between isolates present in Cameroon and two isolates from Mozambique, two geographically distant nations in Africa. Whole genome sequencing can test whether this hypothesis is correct. Our findings set the stage for surveillance and molecular characterization in these areas to elucidate more fully the relationship and disease transmission patterns.

## Introduction

Cholera remains a major public health problem in developing countries, particularly in Africa and Asia, where endemic and epidemic disease continues to devastate vulnerable populations. The etiologic agent of cholera, *V*. *cholerae*, has more than 200 serogroups, differentiated by the O-antigen on the lipopolysaccharide (LPS) of the bacteria’s outer membrane [1]. Of these 200 serogroups, only those that produce cholera toxin (CT) are known to cause epidemic and pandemic disease, primarily serogroups O1 and O139 [2]. *Vibrio* species, and even pathogenic and nonpathogenic *V*. *cholerae* can generally be differentiated using basic biochemical and serological techniques [1]. However, more advanced molecular techniques are needed to differentiate among different pathogenic isolates; which provides crucial information to understand whether distinct isolates cause outbreaks in different geographic areas or whether there are common isolates that spread through wide geographic areas.

As previously described [3], there are a number of molecular methods that have been established for molecular characterization of *V*. *cholerae* isolates, but multi-locus variable-number tandem-repeat analysis (MLVA) allows for differentiation of isolates that are weakly discriminated using other molecular methods. Methods including pulsed-field gel electrophoresis (PFGE), and multi-locus sequence typing (MLST) have limited ability to differentiate among clinical isolates due to the genetic similarity between pathogenic isolates [3]. MLVA examines short DNA sequences that are repeated at a specific locus. The method uses the number of repeats at each specific locus to differentiate between isolates [4] and has shown substantial variation between isolates in a single outbreak [5]. Whole genome sequencing (WGS) is another molecular method that differentiates genetic lineages and in concert with phylogenetic analysis can estimate migration patterns over time and space [6] and may be used in local outbreaks [7].

To date, many of the studies published using MLVA methods to characterize *V*. *cholerae* isolates have focused on the endemic areas of south Asia [4,8]. A study performed in Bangladesh to examine environmental isolates in comparison to clinical isolates demonstrated that the *V*. *cholerae* O1and O139 were endemic in the aquatic environment near Bakerganj [9]. Subsequent MLVA analysis on both clinical and environmental isolates demonstrated that the isolates collected from two outbreak sites, Bakerganj or Mathbaria, were distinct *V*. *cholerae* populations. Additionally, they found that clinical or environmental isolates from a given time period were more likely to have a common genotype than those collected in a subsequent month or time period [3]. In their sample, only a few clinical and environmental isolates had identical genotypes [10]. Further research is warranted to assess the suggested benefits of using MLVA genotypes to determine genetic relatedness during outbreaks, especially in geographic areas such as sub-Saharan Africa where the epidemiology likely differs from that of Bangladesh.

There has been limited research on the molecular characterization of cholera in Africa, and even less research in regards to understanding the molecular epidemiology of cholera in Africa. As the genetic diversity of toxigenic *V*. *cholerae* strains increases; it is increasingly important to understand their relationships and their epidemic potential [11]. One study employed MLVA to characterize clinical isolates from outbreaks beginning in January of 2009 in Kenya. The demonstration of multiple distinct lineages that were also temporally and geographically independent supports the hypothesis that these outbreaks were the result of endemic *V*. *cholerae* rather than imported cases or those spread by travelers [12]. WGS of isolates from the same outbreak and other outbreaks in Kenya revealed that two genetic lineages of *V*. *cholerae* have been circulating in Kenya for ten years and the 2009 outbreak has at least two foci. Recent studies have suggested that the concerted use of both MLVA and WGS for evolutionary relationships and longer term epidemiological typing [13]. Therefore, the continued use of MLVA and WGS for differentiation of clinical cholera isolates as well as any potential environmental isolates may provide further evidence of endemic foci.

In this study, we compared isolates and enriched specimens collected using simplified methods from two recent but geographically distinct outbreaks in Cameroon. We subsequently used MLVA to compare these isolates to ones from recent outbreaks in Mozambique and in the Philippines.

## Methods

### Ethics statement

The Johns Hopkins Bloomberg School of Public Health Institutional Review Board reviewed and approved the study "Sustainable Cholera Surveillance for Cameroon", IRB No. IRB00003981. Written informed consent was obtained from all study participants. Parents or legal guardians of minors provided written informed consent on their behalf. Specimens included in the study from Mozambique were isolated from participants enrolled in the Global Enteric Multicentric Study (GEMS). The GEMS clinical protocol and informed consent were approved by the National Bioethics Committee of Mozambique (CNBS), the ethics committee of the Hospital Clinic of Barcelona and the Institutional Review Board at the University of Maryland. Written informed consent was obtained from the caretaker of each participant prior to initiation of study activities. The ethical statement, study design and population characteristics have been described elsewhere [14,15]. Banked isolates from the Philippines were collected as part of national public health response, stored in the Research Institute for Tropical Medicine, and were provided to Johns Hopkins without any identifiers.

### Clinical surveillance

**Fig 1:**
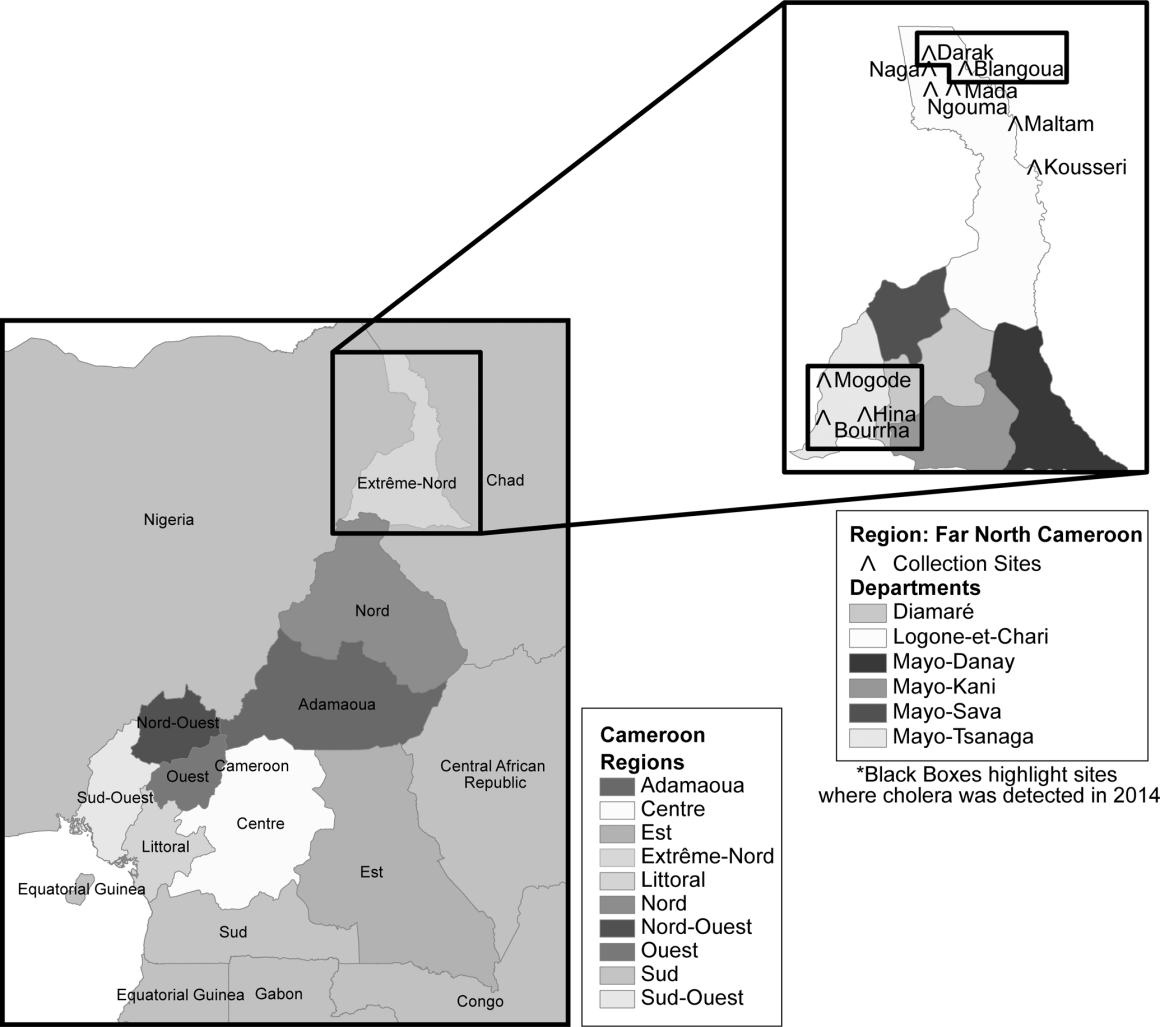
Map of field sites, Far North Cameroon. Clinical isolates from Mozambique were collected from children under five years of age presenting with moderate-to-severe diarrhea.[14]. Clinical isolates from the Philippines were collected during routine surveillance efforts in the national health system. Specimens were collected, tested and confirmed for cholera via classical methods.

Clinical surveillance was initiated in the Far North of Cameroon (FNC) in August 2013. Surveillance was established at 7 seven local health facilities (LHF), in and around Lake Chad: Kousseri, Mada, Ngouma, Maltam, Blangoua, Darak, and Naga (Fig 1). The methodology and surveillance findings during the first year of surveillance have been published previously [16]. In addition to surveillance activities, in June of 2014 the surveillance team was notified of an outbreak outside of the surveillance area in the Bourrha, Mogode and Hina Health Districts. The team was deployed to the outbreak area on repeated occasions between 18 June to 9 August 2014 to assist and apply the study’s simplified field diagnostics to provide rapid diagnosis and confirmation of cases 18 June to 9 August 2014. All consenting subjects with diarrhea provided a fecal specimen for *V*. *cholerae* screening. The shapefiles were obtained from an open-source site, DIVA-GIS[17]. ArcGIS 10.3 (ESRI Inc., USA) was used to analyze the geographical data to produce the maps in Fig 1 [18].

### Laboratory methods

Cameroonian fecal specimens were screened for *V*. *cholerae* O1 and O139 using an enriched dipstick method, detailed in the manufacturer’s package insert (Crystal VC, *Span Diagnostics* Ltd. 173-B, New Industrial Estate, Road No. 6-G, Udhna, Surat—394 210, INDIA). The specimen was tested at the facility of collection via dipstick after enrichment for 6–8 hours at 37°C if available (room temperature between 20–40°C is sufficient) in alkaline peptone water (APW) [19]. APW enriched specimens which tested positive, as well as selected negative clinical specimens were inoculated into Cary-Blair transport media for storage until transport for microbiological confirmation in the central reference laboratory at the Kousseri Health Facility. To evaluate the use of simplified specimen preservation and sample shipping methods, the APW enriched specimen for each stool was also preserved on Whatman 903 filter paper (Whatman 903 Protein Saver Card, GE Healthcare Ltd., Forest Farm, Cardiff, UK) to be tested for *V*. *cholerae* using molecular methods. 1–2 drops of the enriched specimen was aliquoted onto the Whatman filter paper and allowed to air dry; filter papers were stored in individual plastic bags at room temperature until they were sent for DNA extraction and PCR processing.

Inoculated Cary-Blair swabs received at the Kousseri Laboratory for culture confirmation were streaked directly onto thiosulfate citrate bile salt sucrose (TCBS) agar and incubated for 24 hours at 37°C. Immediately after inoculating the first TCBS plate, a pre-labeled APW vial was inoculated with the received swab and incubated for 6 hours at room temperature. After 6 hour incubation, a second TCBS plate is inoculated with the enriched specimen and incubated as before. After the 24 hour incubation, any cholera-like colonies were selected with a sterile loop, re-suspended in 1–2 drops of Phosphate-buffered saline (PBS) and tested via dipstick (Span Diagnostics, Surat, India). All dipstick positive cultures and any cultures considered cholera-suspect, because they demonstrated the morphology of a cholera colony, were preserved in T1N1 agar (1% tryptone and 1% NaCl). Additionally, any dipstick positive cultures or cholera-suspect cultures were selected for filter paper preservation. One colony was selected with a sterile loop, re-suspended in 10 ml of APW and incubated for 6 hours at 37°C. Using a Pasteur pipette, one to two drops of the enriched APW specimen were preserved on Whatman filter paper and allowed to air dry. Filter papers were stored in individual plastic bags at room temperature until DNA extraction and PCR processing were completed.

DNA from isolates for MLVA genotype analysis from the Philippines was preserved and shipped on Whatman filter paper. The isolates were revived from glycerol by streaking TCBS plates and incubating overnight at 37°C. A single colony was then selected to inoculate APW broth with 1% NaCl and then incubated for 6 hours at 37°C. The broth was then boiled for 10 minutes to kill the organisms, and one to two drops of the broth preserved on Whatman filter paper and allowed to air dry. Filter papers were stored in individual plastic bags at room temperature until DNA extraction and PCR processing were completed.

Identification and isolation of *Vibrio* species from diarrheal subject fecal specimens in Mozambique were performed using classic microbiological methods described previously [20]. DNA from purified isolates from Manhica, Mozambique was extracted at the time of molecular analysis.

DNA extractions of filter papers using chelex-100 (BioRad) and subsequent confirmation of *V*. *cholerae* O1 by successful PCR amplification of toxR, ompW, ctxA and rfp genes were performed following described methods [21–24]. The *V*. *cholerae* O1 PCR positive samples were then genotyped at five MLVA loci: VC0147, VC0436-7 (intergenic), VC1650, VCA0171 and VCA0283 [3]. Each locus was amplified using MLVA primers (Table 1) and PCR conditions described previously [3,8]. The presence of amplified products was confirmed by gel electrophoresis. The amplified products were separated using a 3730xl Automatic Sequencer and the size was determined using internal lane standards (LIZ600) with the GeneScan program (all from Applied Biosystems, ABI, Life Technologies, Grand Island, NY) in combination with the formulae in Table 1.

**Table 1:** Primers and formulae for *V*. *cholerae* MLVA.

Primer Name	Sequence	Range	Formula
VC0147-F	TTGTCATGGCTTGGATTTGG	186–224	(x-150)/6
VC0147-R	TET-ACGTGCAGGTTCAACCGTG		
VC0437-F	CGTTAGCATCGAAACTGCTG	265–301	(x-245)/6
VC0437-R	TET-GTTGCCGCCATCACCAGCTTG		
VC1650-F	CTACCAAGCGGCGGTTAAGCTG	370–440	(x-306)/9
VC1650-R	TET-CCGCTAACTGAGTGACCGC		
VCA0171-F	GCTGAAGCCTTTCGCGATCC	316–442	(x-265)/6
VCA0171-R	FAM-AGGCGCCTGATGACGAATCC		
VCA0283-F	AGCCTCCTCAGAAGTTGAG	118–244	(x-95)/6
VCA0283-R	FAM-GGAGGTAGCTACGAATTCTAC		

Alleles were determined by the number of repeats at each locus, and listed in order to generate an isolate genotype: VC0147, VC0437, VC1650, VCA0171, and VCA0283. Therefore the genotype 6-4-6-17-20 indicates 6 repeats at the locus VC0147, 4 at the promoter of VC0437, etc. [3]. Genetic relatedness of the strains was determined using eBURSTv3 (http://eburst.mlst.net). Genotypes were defined as a clonal complex, when the genotypes were related to each other by an allelic change at a single locus.

## Results

A total of 87 *V*. *cholerae* clinical samples were analyzed by MLVA. In total 65 samples from two distinct outbreaks in Cameroon were included: 20 isolates from 20 patients from Bourrha, Hina Health and Mogode Health districts collected in June, 2014; 41 samples (isolates and enriched specimens) from 26 patients collected in Darak in October 2014; 4 samples (isolates and enriched specimens) from 2 patients collected in Blangoua in October 2014. In addition, 14 isolates from 14 patients in outbreaks in the Philippines were included. Of these, 4 were from Lopez, Quezon in December 2012; 3 from Sinawal, General Santos City in April and May 2013; 3 from T’boli, South Cotabato in May 2013; and 4 from the 2014 outbreak in Davao del Sur. Finally, 8 isolates from 8 patients were from Manhica, Mozambique, 5 from January 2008 and 1 each from February and March 2009 (Table 2).

**Table 2:** *V*. *cholerae* specimen genotypes and MLVA group.

Original ID	Specimen Type	Location, Year	VC0147	VC0437	VC1650	VCA0171	VCA0283	MLVA Group
15B_Cam	Isolate	Bourrha, CMR*; June 2014	6	4	6	17	20	1
300205 (VC Ogawa)_F/4	Isolate	Manhica, MOZ Jan 2008	7	4	2	13	14	Singleton
003B PHIL.	Isolate	Sinawal,General Santos, PHL^ᵮ^; April 2013	7	9	9	7	21	Singleton
300043 (VC Ogawa) _F/5	Isolate	Manhica, MOZ Jan 2008	8	4	6	18	21	5
300208 (VC Ogawa)_F/6	Isolate	Manhica, MOZ Jan 2008	8	4	6	18	21	5
300209 (VC Ogawa)_F/7	Isolate	Manhica, MOZ Jan 2008	8	4	6	19	21	5
5B_Cam	Isolate	Bourrha, CMR; June 2014	9	4	6	17	19	1
12B_Cam	Isolate	Bourrha, CMR; June 2014	9	4	6	17	19	1
6B_Cam	Isolate	Bourrha, CMR; June 2014	9	4	6	17	19	1
25B_Cam	Isolate	Bourrha, CMR; June 2014	9	4	6	18	20	1
14B_Cam	Isolate	Bourrha, CMR; June 2014	9	4	6	17	20	1
21B_Cam	Isolate	Bourrha, CMR; June 2014	9	4	6	17	20	1
7B_Cam	Isolate	Bourrha, CMR; June 2014	9	4	6	17	20	1
20B_Cam	Isolate	Bourrha, CMR; June 2014	9	4	6	17	20	1
23B_Cam	Isolate	Bourrha, CMR; June 2014	9	4	6	17	20	1
22B_Cam	Isolate	Bourrha, CMR; June 2014	9	4	6	17	20	1
11B_Cam	Isolate	Bourrha, CMR; June 2014	9	4	6	17	20	1
24B_Cam	Isolate	Bourrha, CMR; June 2014	9	4	6	17	20	1
26B_Cam	Isolate	Bourrha, CMR; June 2014	9	4	6	17	20	1
29B_Cam	Isolate	Bourrha, CMR; June 2014	9	4	6	17	20	1
28B_Cam	Isolate	Bourrha, CMR; June 2014	9	4	6	17	20	1
4B_Cam	Isolate	Bourrha, CMR; June 2014	9	4	6	17	21	1
302015 (VC Ogawa)_F/1	Isolate	Manhica, MOZ Feb 2009	9	4	6	18	23	1
27B_Cam	Isolate	Bourrha, CMR; June 2014	9	4	6	17	23	1
014B PHIL.	Isolate	Sinawal,General Santos, PHL; April 2013	11	4	1	13	14	Singleton
013B PHIL.	Isolate	Lopez, Quezon, PHL; Dec2012	11	9	10	17	20	2
004B PHIL.	Isolate	T’boli, South Cotabato, PHL; May 2013	11	9	10	17	21	2
017B PHIL.	Isolate	Lopez, Quezon, PHL; Dec 2012	11	9	10	14	24	Singleton
011B PHIL.	Isolate	Davao del Sur, PHL; July 2014	12	9	8	22	27	4
005B PHIL.	Isolate	Davao del Sur, PHL; July 2014	12	9	9	22	27	4
012B PHIL.	Isolate	Davao del Sur, PHL; July 2014	12	9	9	23	27	4
010B PHIL.	Isolate	Davao del Sur, PHL; July 2014	12	9	9	22	27	4
007B PHIL.	Isolate	T’boli, South Cotabato, PHL; May 2013	12	9	10	17	21	2
009B PHIL.	Isolate	T’boli, South Cotabato, PHL; May 2013	12	9	10	17	22	2
600070-DN	Isolate	Darak,CMR; Oct 2014	8	4	7	10	25	Singleton
600068-DP	Isolate	Darak,CMR; Oct 2014	9	4	6	12	25	3
600059-DP	Isolate	Darak,CMR; Oct 2014	9	4	6	14	25	3
600052-DP	Isolate	Darak,CMR; Oct 2014	9	4	6	14	25	3
600052-DR	Enriched	Darak,CMR; Oct 2014	9	4	6	14	25	3
600066-DR	Enriched	Darak,CMR; Oct 2014	9	4	6	14	25	3
600064-DR	Enriched	Darak,CMR; Oct 2014	9	4	6	14	25	3
600072-DP	Isolate	Darak,CMR; Oct 2014	9	4	6	14	25	3
600058-DP	Isolate	Darak,CMR; Oct 2014	9	4	6	14	25	3
600066-DP	Isolate	Darak,CMR; Oct 2014	9	4	6	14	25	3
600071-DP	Isolate	Darak,CMR; Oct 2014	9	4	6	14	25	3
600064-DP	Isolate	Darak,CMR; Oct 2014	9	4	6	14	25	3
600059-DR	Enriched	Darak,CMR; Oct 2014	9	4	6	14	25	3
600058-DR	Enriched	Darak,CMR; Oct 2014	9	4	6	14	25	3
600071-DR	Enriched	Darak,CMR; Oct 2014	9	4	6	14	25	3
500289-APW	Enriched	Blangoua,CMR; Oct 2014	9	4	6	15	25	3
500289-culture	Enriched	Blangoua,CMR; Oct 2014	9	4	6	15	25	3
600070-DR	Enriched	Darak,CMR; Oct 2014	9	4	6	16	25	3
600057-DP	Isolate	Darak,CMR; Oct 2014	9	4	6	16	25	3
600048-DP	Isolate	Darak,CMR; Oct 2014	9	4	6	16	25	3
500291-culture	Isolate	Blangoua,CMR; Oct 2014	9	4	6	16	25	3
600046-DP	Isolate	Darak,CMR; Oct 2014	9	4	6	16	25	3
600060-DP	Isolate	Darak,CMR; Oct 2014	9	4	6	16	25	3
600055-DR	Enriched	Darak,CMR; Oct 2014	9	4	6	16	25	3
600041-DP	Isolate	Darak,CMR; Oct 2014	9	4	6	16	25	3
600054-DP	Isolate	Darak,CMR; Oct 2014	9	4	6	16	25	3
600069-DP	Isolate	Darak,CMR; Oct 2014	9	4	6	16	25	3
600055-DP	Isolate	Darak,CMR; Oct 2014	9	4	6	16	25	3
600067-DP	Isolate	Darak,CMR; Oct 2014	9	4	6	16	25	3
600065-DR	Enriched	Darak,CMR; Oct 2014	9	4	6	16	25	3
600050-DP	Isolate	Darak,CMR; Oct 2014	9	4	6	16	25	3
600057-DR	Enriched	Darak,CMR; Oct 2014	9	4	6	16	25	3
600040-DP	Isolate	Darak,CMR; Oct 2014	9	4	6	16	25	3
600060-DR	Enriched	Darak,CMR; Oct 2014	9	4	6	16	25	3
600061-DR	Enriched	Darak,CMR; Oct 2014	9	4	6	16	25	3
600065-DP	Isolate	Darak,CMR; Oct 2014	9	4	6	16	25	3
500291-APW	Enriched	Blangoua,CMR; Oct 2014	9	4	6	16	25	3
600045-DP	Isolate	Darak,CMR; Oct 2014	9	4	6	16	25	3
600047-DP	Isolate	Darak,CMR; Oct 2014	9	4	6	16	25	3
600061-DP	Isolate	Darak,CMR; Oct 2014	9	4	6	16	25	3
600069-DR	Enriched	Darak,CMR; Oct 2014	9	4	6	16	25	3
600043-DP	Isolate	Darak,CMR; Oct 2014	9	4	6	16	25	3
600053-DR	Isolate	Darak,CMR; Oct 2014	9	4	6	16	25	3
600067-DR	Enriched	Darak,CMR; Oct 2014	9	4	6	16	25	3
600068-DR	Enriched	Darak,CMR; Oct 2014	9	4	6	16	25	3
30B_Cam	Isolate	Bourrha, CMR; June 2014	9	4	6	17	23	1
1B_Cam	Isolate	Bourrha, CMR; June 2014	9	4	6	17	15	1
300215 (VC Ogawa)_E/10	Isolate	Manhica, MOZ Feb 2008	8	4	6	18	21	5
302029 (VC Ogawa)_F/2	Isolate	Manhica, MOZ Mar 2009	9	4	6	18	24	1
300055 (VC Ogawa)_F/8	Isolate	Manhica, MOZ Jan 2008	12	4	7	12	14	Singleton
006B PHIL.	Isolate	Lopez, Quezon, PHL; Dec2012	12	9	9	22	27	4
018B PHIL.	Isolate	Lopez, Quezon, PHL; Dec2012	11	9	10	15	13	Singleton
008B PHIL.	Isolate	Sinawal,General Santos, PHL; May 2013	12	9	10	17	21	2

CMR* = Cameroon

MOZ ^ᶲ^ = Mozambique

PHL^ᵮ^ = Philippines

DNA was successfully analyzed from all 87 samples. Of these 87 samples, 16 were analyzed in two forms: a pure culture isolate and an APW enriched specimen from the stool (Table 3). One dipstick positive-enriched specimen was paired with a dipstick negative isolate (ID 600070), and this was confirmed by PCR and MLVA, demonstrating that the isolate selected was a *V*. *cholerae* non-O1 specimen. One dipstick positive-enriched specimen did not have a matching isolate preserved on filter paper at the time of analysis. Of the 16 pure isolate-enriched specimen pairs that had identical dipstick results (i.e. positive for *V*. *cholerae* O1); the genotypes from the cultured isolates were identical to those of the enriched specimens in 15 (94%). The enriched specimen-isolate pair that differed (600078), did so at a single small chromosome locus.

**Table 3:** Patient specimen; quantity and type by country.

Country	Crude Specimens	Isolates	Matched Crude-Isolate Pairs	Patients	Total
Bourrha, Hina, Mogode, Cameroon	0	20	0	20	20
Darak, Cameroon	16	25	14*	26	41
Blangoua, Cameroon	2	2	2	2	4
Philippines	0	14	0	14	14
Mozambique	0	8	0	8	8
**Total**	18	69	16	69	87

*16 crude specimens obtained, but 2 did not have V. cholerae O1 isolates for MLVA comparison

When all five loci were considered, alleles were more likely to differ at the small chromosome loci (VCAx). The number of distinct alleles among the isolates at loci VC0147, VC0437, VC1650, VCA0171, and VCA0283 were 6, 2, 7, 12 and 11, respectively (Table 4). There were 29 distinct genotypes among the 87 specimens analyzed; 5 clonal complexes and 5 singletons were identified when the genotypes were analyzed using eBURST.

**Table 4:** Number and percentage of initial *V*. *cholerae* O1 isolates differing at each loci.

No. of V. cholerae O1 Specimens	No. of isolates differing at each loci				
	Large-chromosome loci			Small-chromosome loci	
	VC0147	VC0437	VC1650	VCA0171	VCA0283
Overall (87)	6	2	7	12	11
Cameroon Isolate & Enriched Specimens (65) **	3	1	2	7	6
Matching Isolate -enriched specimen pairs (16)**	0	0	0	1 (6.7)^¥^	0
Phillipines (14)	3	2	4	7	7
Mozambique (8)	4	1	3	4	4

^¥^ One isolate (600068) differed from its enriched specimen genotype at the 4^th^ locus

**Fig 2:**
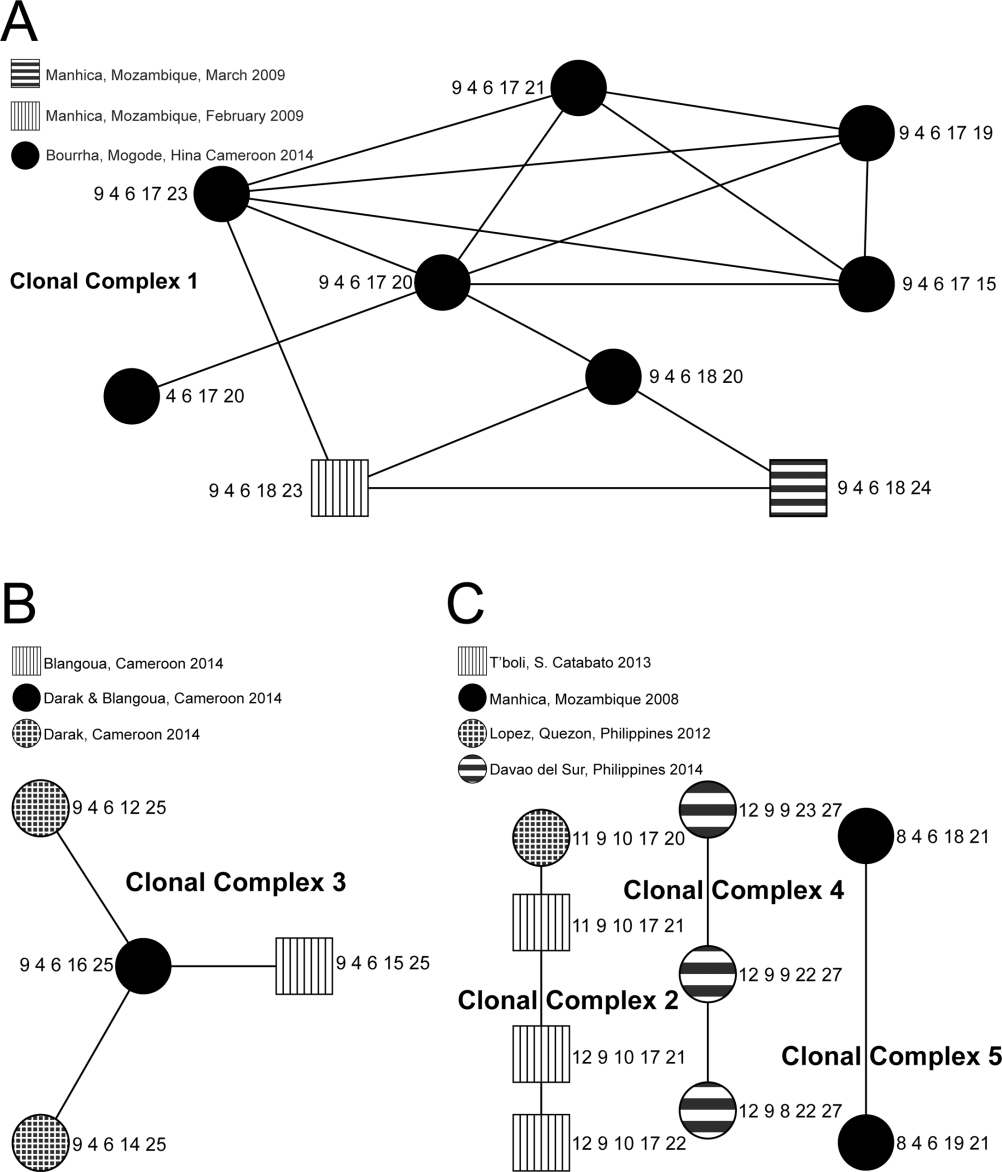
A. Clonal Complex 1. B. Clonal Complex 3. C. Clonal Complexes 2, 4 & 5.

The Cameroon genotypes belonged to two clonal complexes, 1 & 3 (Fig 2) and one singleton was unrelated to any of the others. Clonal complex 1 contained 9 different genotypes from 20 isolates from the same outbreak in the Bourrha districts in June 2014 and from two isolates from Manhica, Mozambique from February and March 2009. The center of the clonal complex is the genotype with the largest number of single-locus variants (SLVs) [25]. In clonal complex 1, this genotype (9-4-6-17-20) was present in 11 Bourrha clinical isolates. The diverging genotypes included clinical isolates from the same districts in Cameroon as well as two additional genotypes (9-4-6-18-23; 9-4-6-18-24) found in Mozambican clinical isolates.

Clonal complex 3 contains 4 genotypes from 45 isolates and enriched specimens. The center of the clonal complex is a genotype (9-4-6-16-25) that was observed in 28 isolates and enriched specimens from the outbreak on the island of Darak in October 2014 and one clinical isolate from the Blangoua District Medical Center (Mada Health District) in October 2014. Three genotypes radiated from this founder, the first two genotypes 9-4-6-14-25 and 9-4-6-12-25 were from the Darak Outbreak and the third 9-4-6-15-25 was from the Blangoua District Medical Center (Mada Health District).

The Philippine isolates comprised clonal complexes 2 & 4 and 3 singletons. The isolates from the outbreaks in Lopez, Quezon in 2012 and T’boli, South Cotabato in 2013 clustered in clonal complex 2 comprised of 4 genotypes. While the isolates from the outbreak in July 2014 in Davao del Sur comprised clonal complex 4 which has 3 genotypes.

Of the eight specimens from Mozambique included in this analysis; 4 isolates clustered together into 2 genotypes in clonal complex 5, 2 genotypes were in clonal complex 1 and 2 were singletons (Table 2).

## Discussion

The results of this study showed that using simplified laboratory diagnostics, including filter paper for specimen preservation, in remote and unstable field settings allowed for easy sample storage and transport in spite of difficult settings. When this simplified DNA preservation method was used, we were able to molecularly characterize isolates in an on-going outbreak. These rapid results can provide key stakeholders in the country information regarding disease transmission patterns to allow more proactive planning regarding interventions to prevent further spread.

The comparison of the genotypes of 16 cultured *V*. *cholerae* O1 isolates to enriched specimens from APW enrichment preserved on filter paper demonstrated that there were few differences in the genotypic results between the two methods of preservation. The enriched specimen-isolate pair that differed when compared according to MLVA genotype varied only at the most variable locus and were still grouped into the same clonal complex. In settings where culture of *V*. *cholerae* is not possible, these simplified methods offer a low cost, low maintenance alternative to characterize *Vibrio* strains. The results of the genetic analyses did not suggest significant genetic diversity within Cameroon. While specimens from the two Cameroonian outbreaks in 2014 form distinct eBURST clonal complexes, differing at more than 1 loci, all of the genotypes are identical at the three large chromosome loci (9-4-6-X-X). Due to the difference in the 2 small chromosome loci and the limited number of samples for comparison, it is not apparent whether the related strains were spread by travelers in the region or whether this strain persists in the FNC on a small scale, and conditions were favorable in 2014 for its spread. Interestingly, two isolates from Manhica, Mozambique in 2009 were identified as being related to the strains present in the Bourrha outbreak in Cameroon. Whether this is convergence of MLVA genotypes or a phylogenetic similarity will require whole genome sequencing.

The outbreaks in 2014 in Philippines are clearly distinct from those in Cameroon and Mozambique. However, the identification of two clonal complexes demonstrates that the 2014 outbreak in Davao del Sur is distinct from the 2012 and 2013 outbreaks. The use of filter paper preservation of isolates was used to enable shipment of DNA only for timely molecular characterization of the 2014 strains in comparison to those from previous years.

There were limitations in conducting the study, including the difficulty in obtaining data and samples consistently due to concerns about security and safety of the staff in the outbreak areas in the FNC in 2014. Although working in difficult circumstances, the team was able to train the local health staff using our simplified diagnostics techniques for confirming cholera. The local insurgent group made it unsafe for the team to work in the area for long periods. The ability to provide timely molecular results is dependent on the connection to field site labs with molecular capabilities or collaboration with external partners with such facilities. While this is not the case in most African settings, we hope that the simplicity of filter paper for storage and shipping will increase collaborative relationships to further understand the genetic relatedness of circulating cholera strains.

This study demonstrates that simple and low-cost lab methods can be utilized in even the most vulnerable and resource limited settings and allow for molecular characterization of cholera outbreaks in a rapid and timely manner. The molecular data gathered in this study were promptly presented to the Ministry of Health of Cameroon to inform them that the strains in the two areas of Cameroon were similar and did not appear to represent the emergence of a new strain. With experience, these rapid molecular methods may help to track transmission patterns and aid the outbreak response.

The strains present in 2014 outbreaks in Southeast Asia are distinct from those in Africa. Interestingly, we may have detected a relationship between strains present in the 2014 outbreaks in Cameroon and those isolated from Mozambique in 2009, two geographically distant nations in Africa. This finding and the fact that the isolates in the two outbreaks at distinct areas of FNC were similarly related warrants continued surveillance molecular characterization in these areas to elucidate more fully the relationship and disease transmission patterns.

## References

[1] Chatterjee SN, Chaudhuri K (2003) Lipopolysaccharides of Vibrio cholerae. I. Physical and chemical characterization. Biochim Biophys Acta 1639: 65–79. 14559113 10.1016/j.bbadis.2003.08.004

[2] Chen Y, Johnson JA, Pusch GD, Morris JG Jr., Stine OC (2007) The genome of non-O1 Vibrio cholerae NRT36S demonstrates the presence of pathogenic mechanisms that are distinct from those of O1 Vibrio cholerae. Infect Immun 75: 2645–2647. 17283087 10.1128/IAI.01317-06 PMC1865779

[3] Kendall EA, Chowdhury F, Begum Y, Khan AI, Li S, et al (2010) Relatedness of Vibrio cholerae O1/O139 isolates from patients and their household contacts, determined by multilocus variable-number tandem-repeat analysis. J Bacteriol 192: 4367–4376. 10.1128/JB.00698-10 20585059 PMC2937383

[4] Danin-Poleg Y, Cohen LA, Gancz H, Broza YY, Goldshmidt H, et al (2007) Vibrio cholerae strain typing and phylogeny study based on simple sequence repeats. J Clin Microbiol 45: 736–746. 17182751 10.1128/JCM.01895-06 PMC1829105

[5] Rebaudet S, Mengel MA, Koivogui L, Moore S, Mutreja A, et al (2014) Deciphering the origin of the 2012 cholera epidemic in Guinea by integrating epidemiological and molecular analyses. PLoS Negl Trop Dis 8: e2898 10.1371/journal.pntd.0002898 24901522 PMC4046952

[6] Kiiru J, Mutreja A, Mohamed AA, Kimani RW, Mwituria J, et al (2013) A study on the geophylogeny of clinical and environmental Vibrio cholerae in Kenya. PLoS One 8: e74829 10.1371/journal.pone.0074829 24066154 PMC3774669

[7] Azarian T, Ali A, Johnson JA, Mohr D, Prosperi M, et al (2014) Phylodynamic analysis of clinical and environmental Vibrio cholerae isolates from Haiti reveals diversification driven by positive selection. MBio 5. 10.1128/mBio.01824-14 PMC4278535 25538191

[8] Ghosh R, Nair GB, Tang L, Morris JG, Sharma NC, et al (2008) Epidemiological study of Vibrio cholerae using variable number of tandem repeats. FEMS Microbiol Lett 288: 196–201. 10.1111/j.1574-6968.2008.01352.x 18811655

[9] Alam M, Hasan NA, Sadique A, Bhuiyan NA, Ahmed KU, et al (2006) Seasonal cholera caused by Vibrio cholerae serogroups O1 and O139 in the coastal aquatic environment of Bangladesh. Appl Environ Microbiol 72: 4096–4104. 16751520 10.1128/AEM.00066-06 PMC1489596

[10] Stine OC, Alam M, Tang L, Nair GB, Siddique AK, et al (2008) Seasonal cholera from multiple small outbreaks, rural Bangladesh. Emerg Infect Dis 14: 831–833. 10.3201/eid1405.071116 18439375 PMC2600222

[11] Bhuiyan NA, Nusrin S, Ansaruzzaman M, Islam A, Sultana M, et al (2012) Genetic characterization of Vibrio cholerae O1 strains isolated in Zambia during 1996–2004 possessing the unique VSP-II region of El Tor variant. Epidemiol Infect 140: 510–518. 10.1017/S0950268811000926 21676349

[12] Mohamed AA, Oundo J, Kariuki SM, Boga HI, Sharif SK, et al (2012) Molecular epidemiology of geographically dispersed Vibrio cholerae, Kenya, January 2009-May 2010. Emerg Infect Dis 18: 925–931. 10.3201/eid1806.111774 22607971 PMC3358164

[13] Lam C, Octavia S, Reeves PR, Lan R (2012) Multi-locus variable number tandem repeat analysis of 7th pandemic Vibrio cholerae. BMC Microbiol 12: 82 10.1186/1471-2180-12-82 22624829 PMC3438101

[14] Kotloff KL, Nataro JP, Blackwelder WC, Nasrin D, Farag TH, et al (2013) Burden and aetiology of diarrhoeal disease in infants and young children in developing countries (the Global Enteric Multicenter Study, GEMS): a prospective, case-control study. Lancet 382: 209–222. 10.1016/S0140-6736(13)60844-2 23680352

[15] Nhampossa T, Mandomando I, Acacio S, Quinto L, Vubil D, et al (2015) Diarrheal Disease in Rural Mozambique: Burden, Risk Factors and Etiology of Diarrheal Disease among Children Aged 0–59 Months Seeking Care at Health Facilities. PLoS One 10: e0119824 10.1371/journal.pone.0119824 25973880 PMC4431848

[16] Debes AK, Ateudjieu J., Guenou E., Ebile W., Sonkoua I.T., Njimbia A.C., Steinwald P., Ram M., Sack D.A. (In Press) Clinical and Environmental Surveillance for *Vibrio cholerae* in Resource Contstrained Areas: Application during a one year surveillance in the Far North Region of Cameroon. Am J Trop Med Hyg. 10.4269/ajtmh.15-0496 PMC4775888 26755564

[17] Hijmans RJ, Guarino, L., Bussink, C., Mathur, P., Cruz, M., Barrentes, I (2015) DIVA-GIS 7.5.

[18] ArcGIS (2015). 10.0 ed. Redlands, California: Esri.

[19] Debes AC, S; Sack, D.A. (2015) Manual for Detecting Vibrio cholerae O1 from Fecal Samples using an Enriched Dipstick Assay—A Low-Cost, Simplified Method of Confirming Cholera. Baltimore, MD.

[20] Panchalingam S, Antonio M, Hossain A, Mandomando I, Ochieng B, et al (2012) Diagnostic microbiologic methods in the GEMS-1 case/control study. Clin Infect Dis 55 Suppl 4: S294–302. 10.1093/cid/cis754 23169941 PMC3502308

[21] Kain KC, Lanar DE (1991) Determination of genetic variation within Plasmodium falciparum by using enzymatically amplified DNA from filter paper disks impregnated with whole blood. J Clin Microbiol 29: 1171–1174. 1864936 10.1128/jcm.29.6.1171-1174.1991 PMC269964

[22] Nandi B, Nandy RK, Mukhopadhyay S, Nair GB, Shimada T, et al (2000) Rapid method for species-specific identification of Vibrio cholerae using primers targeted to the gene of outer membrane protein OmpW. J Clin Microbiol 38: 4145–4151. 11060082 10.1128/jcm.38.11.4145-4151.2000 PMC87555

[23] Hoshino K, Yamasaki S, Mukhopadhyay AK, Chakraborty S, Basu A, et al (1998) Development and evaluation of a multiplex PCR assay for rapid detection of toxigenic Vibrio cholerae O1 and O139. FEMS Immunol Med Microbiol 20: 201–207. 9566491 10.1111/j.1574-695X.1998.tb01128.x

[24] Bauer A, Rorvik LM (2007) A novel multiplex PCR for the identification of Vibrio parahaemolyticus, Vibrio cholerae and Vibrio vulnificus. Lett Appl Microbiol 45: 371–375. 17897378 10.1111/j.1472-765X.2007.02195.x

[25] Rashed SM, Azman AS, Alam M, Li S, Sack DA, et al (2014) Genetic variation of Vibrio cholerae during outbreaks, Bangladesh, 2010–2011. Emerg Infect Dis 20: 54–60. 10.3201/eid2001.130796 24377372 PMC3884724

