# Correction: Evaluation in Cameroon of a Novel, Simplified Methodology to Assist Molecular Microbiological Analysis of *V*. *cholerae* in Resource-Limited Settings

**DOI:** 10.1371/journal.pntd.0004537

**Published:** 2016-03-08

**Authors:** Amanda K. Debes, Jerome Ateudjieu, Etienne Guenou, Anna Lena Lopez, Mark Philip Bugayong, Pearl Joy Retiban, Marcelino Garrine, Inacio Mandomando, Shan Li, O. Colin Stine, David A. Sack

The third author’s name is spelled incorrectly. The correct name is Etienne Guenou. The correct citation is: Debes AK, Ateudjieu J, Guenou E, Lopez AL, Bugayong MP, Retiban PJ, et al. (2016) Evaluation in Cameroon of a Novel, Simplified Methodology to Assist Molecular Microbiological Analysis of *V*. *cholerae* in Resource-Limited Settings. PLoS Negl Trop Dis 10(1): e0004307. doi:10.1371/journal.pntd.0004307
