# Correction: Evaluation in Cameroon of a Novel, Simplified Methodology to Assist Molecular Microbiological Analysis of *V*. *cholerae* in Resource-Limited Settings

**DOI:** 10.1371/journal.pntd.0004573

**Published:** 2016-03-21

**Authors:** Amanda K. Debes, Jerome Ateudjieu, Etiene Guenou, Anna Lena Lopez, Mark Philip Bugayong, Pearl Joy Retiban, Marcelino Garrine, Inacio Mandomando, Shan Li, O. Colin Stine, David A. Sack

There is an error in Fig 2. The authors have provided a corrected a version here.

**Fig 2:**
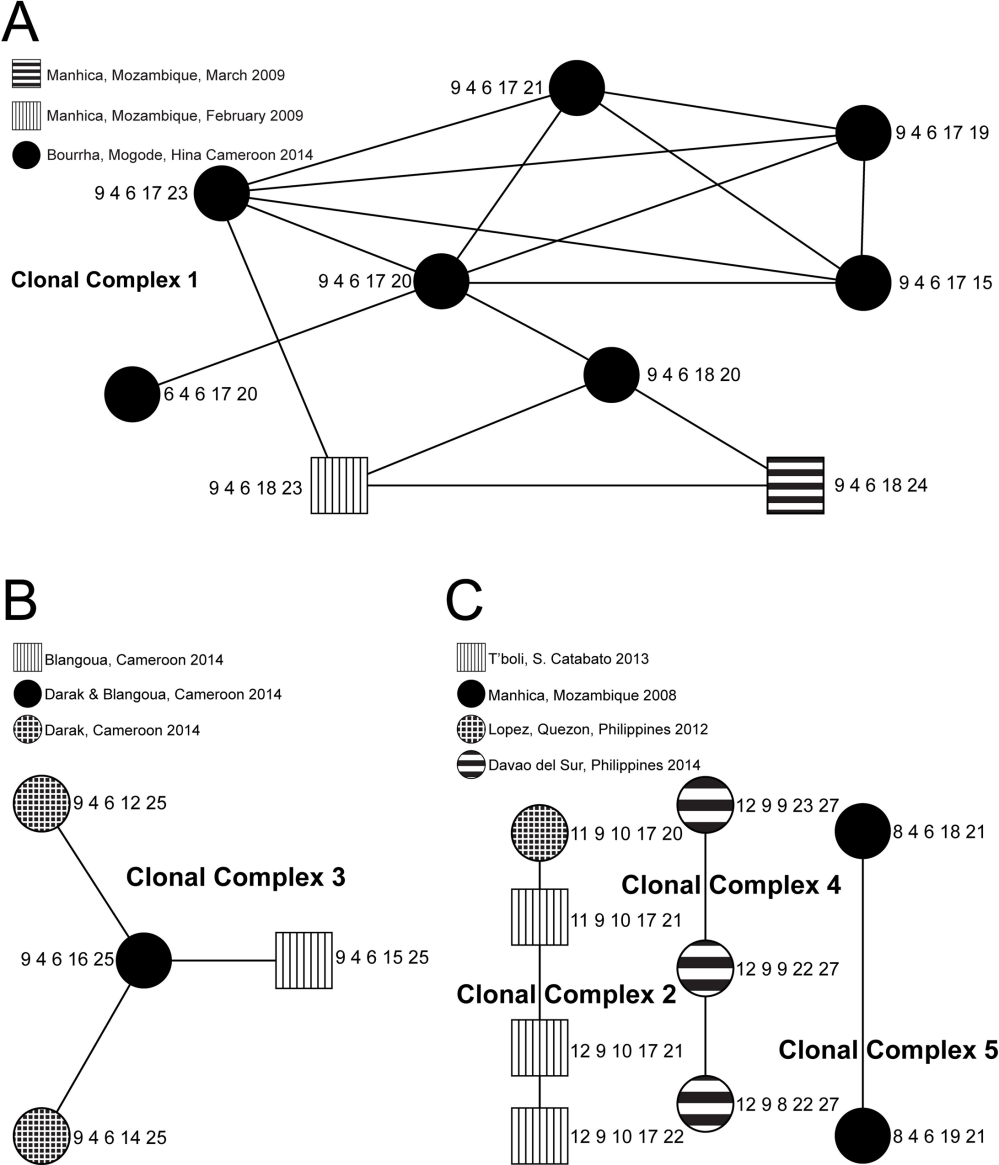
A. Clonal Complex 1. B. Clonal Complex 3. C. Clonal Complexes 2, 4 & 5.
